# Development of a new biological dressing: the modified cross-linking of Chitosan derived from cuttlefish (*Sepia pharaonis*) by-products as an effective agent for promoting cell migration

**DOI:** 10.1186/s40643-025-01002-3

**Published:** 2026-01-27

**Authors:** Ping-Hsiu Huang, Yu-Wei Chen, Jing-Huei Zeng, Bo-Heng Li, Ya-Ting Chen, Shu-Ling Hsieh, Ming-Kuei Shih, Chih-Yao Hou

**Affiliations:** 1 Department of Food Science, College of Life Science, National Chiayi University, No.300 Syuefu Rd, Chiayi City, 600355 Taiwan; 2 Department of Food Science and Biotechnology, National Chung Hsing University, Taichung, 40227 Taiwan; 3 Department of Pediatrics, Kaohsiung Chang Gung Memorial Hospital, Kaohsiung, 83301 Taiwan; 4 Department of Seafood Science, College of Hydrosphere, National Kaohsiung. University of Science and Technology, Kaohsiung, 81157 Taiwan; 5 Graduate Institute of Food Culture and Innovation, National Kaohsiung University of Hospitality and Tourism, Kaohsiung, 812301 Taiwan

**Keywords:** Biomaterial, Gene expression, HaCaT cell lines, Matrix metalloproteinases, Wound healing

## Abstract

This study aimed to extract chitosan (CS) from cuttlefish (*Sepia pharaonis*) bones (CB) and then chemically modify it to produce carboxymethyl chitosan (CMC). Marine cuttlefish skin collagen peptide (MCP) was then cross-linked with CMC to form a novel CMC-MCP complex. The physicochemical properties and biological effects of CS, CMC, MCP, and CMC-MCP were evaluated using human keratinocyte (HaCaT) cell lines. All materials showed cytotoxicity at high concentrations (100–1600 µg/mL), negatively affecting cell viability. At a lower concentration of 50 µg/mL, the materials were used to assess cell migration. Among them, the CMC-MCP complex significantly promoted cell migration. Additionally, CMC-MCP treatment led to increased expression levels of matrix metalloproteinases (MMP-2 and 9) and tissue inhibitors of metalloproteinases (TIMP-1 and 2), which are key regulators in the wound healing process. These findings suggest that the CMC-MCP complex has potential as an economical, safe, and effective biological dressing for promoting wound healing. Further studies are recommended to explore its interaction with other healing-related factors, such as nutrients and growth factors, to better understand its influence on various stages of tissue repair.

**Graphical abstract:**

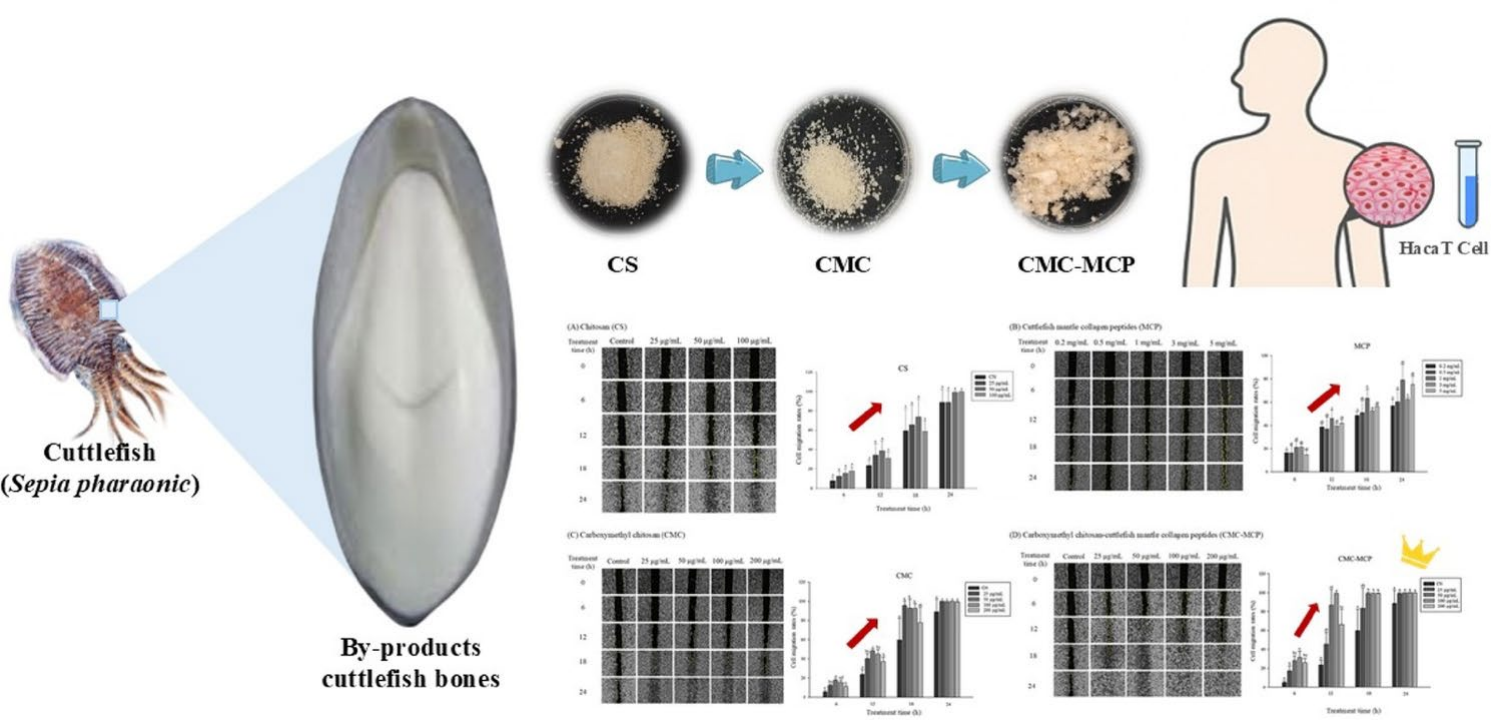

## Introduction

Skin injuries or burn wounds caused by invasive surgeries, accidents, fire, boiling oil, steam, boiling water, chemical substances, diabetes, etc., might be experienced by individuals worldwide (Dugam et al. 2024). Specifically, wound healing depends on the size, depth, and severity of the damage to the epidermis and dermis; nonetheless, a regular skin wound heals within 1–12 weeks (Nqoro et al. 2024; Rahman et al. 2024; Sanjarnia et al. 2024). It is imperative to provide proper wound care regardless of the severity since neglecting wounds can result in escalated treatment costs, disability (amputation), and even death in cases of severe injuries (Dugam et al. 2024; Sanjarnia et al. 2024; Rezvani Ghomi et al. 2019; Zhou et al. 2021). However, modern wound dressings emerged during the 20th century due to advancements in science and medicine. These wound care products are designed to address the intricate stages of wound healing, encompassing hemostasis, inflammation, cellular processes (adhesion, migration, proliferation), scabbing, and scarring (Dugam et al. 2024; Barzegar et al. 2024; Berry et al. 2024). The global advanced wound care market is estimated to be worth more than USD 33.6 billion by 2030, representing a compound annual growth rate (CAGR) of 15.70% (Godoi et al. 2024). Within this, skin tissue-engineered products are projected to grow to USD 3.2 billion (Godoi et al. 2024). Skin tissue-engineered products (without hair follicles, exocrine glands, or sensory nerves) can effectively stop bleeding, promote wound healing, and reduce wound healing inhibition, inflammation reactions, and subsequent scarring (Godoi et al. 2024). Notably, CS (a biopolymer, N-deacetylation from chitin) hydrogel exhibits biocompatibility, biodegradability, affinity for biomolecules, antibiosis, enhanced mechanical strength, waterproof performance, and wound-healing activity while closely simulating the three-dimensional structures of natural tissues (Barzegar et al. 2024; Hu et al. 2019; Ciftci and Özarslan 2024; Rajinikanth et al. 2024; Shang et al. 2024; Singh et al. 2024; Elumalai et al. 2025). Consequently, it has emerged as a promising biomaterial in tissue engineering (Barzegar et al. 2024; Rajinikanth et al. 2024), while CS-related modified polymers (such as CMC) continue to be developed for various functions and applications, playing a central role (Sanjarnia et al. 2024; Shang et al. 2024; Medeiros Borsagli 2024). In parallel, wound dressings should continue to advance towards more sophisticated, innovative, and cost-effective approaches, considering the increasing population aging, shorter hospital stays, and the persistent rise in chronic ailments such as diabetes-related chronic wounds (Sanjarnia et al. 2024; Medeiros Borsagli 2024; Baran 2024; Vyver and Idensohn 2022).

Moreover, Lima-Junior et al. (2019) in the surgical use of treated tilapia skin (subjected to a process of chemical sterilization, glycerolization, and irradiation, with confirmation of the bacterial and fungal contents) as a xenograft in the innovative treatment of gunpowder blasts with partial thickness burns. The same authors reported that the biomaterial exhibited excellent adherence to the wound, necessitated no dressing changes, and demonstrated no adverse effects during therapy. However, by day 12 of treatment, the fish skin started to desiccate and harden, leading to its detachment from the patient’s limb. Widely recognized, the migratory impact of associated cells (such as fibroblasts and HaCaT) plays a vital role in accelerating the phase of cell division during wound healing and aiding in wound closure through contraction (Lima-Junior et al. 2019; Kamarazaman et al. 2022). In another clinical study, Lima Júnior et al. (2021) reported that treatment with glycerolized Nile tilapia fish skin in a trial group of 57 patients demonstrated exceptional adherence to the wound bed, effectively preventing infection while minimizing the necessity for dressing changes. The authors also indicated that the application of fish skin demonstrated enhanced wound healing (9.7 ± 0.6 days) and improved pain management in burn patients compared to conventional treatment methods (10.2 ± 0.9 days) while offering a cost-effective alternative with a minimum reduction of 42.1% in healthcare expenses. Despite their efficacy in enhancing wound quality for subsequent skin grafting, biological dressings cannot serve as a permanent substitute for skin due to immunological factors; they are employed temporarily solely to facilitate re-epithelialization of the wound (Garfein and Orgill 2003; Wang et al. 2018). The irreversible burden of rejection and immunosuppression imposed by organ transplantation cannot be effectively preempted, while immunosuppression can only be administered during the later stages of graft rejection or injury (Urie et al. 2024).

Consequently, biomaterials with potential as wound dressings are further processed into collagen-based hydrogels to enhance their advantages, enabling the dressings to create a moist environment for wound healing and to combine with other actives for improved effectiveness (Zimba et al. 2024). Collagen exhibits potential as a biomaterial for wound dressings, as it can enhance wound healing by promoting cellular growth (adhesion and proliferation), demonstrating advantageous physical and mechanical properties, displaying minimal immunogenicity, and being biocompatible and biodegradable (Chen et al. 2019a, b). Interestingly, Luo et al. (2023) reported that the cellular dermal matrix of fish skin exhibited the type I collagen network, low immunogenicity, and good biocompatibility properties. Vacuum freeze-drying enabled the formation of porous sponges, which contributed to the absorption of more blood and tissue leachate, thereby facilitating hemostasis (Luo et al. 2023). Miranda et al. (2024) reported that cellular type III collagen fibers in rat scar tissue dominate the process’s inflammatory and proliferative phases by serving as scaffolds and supporting type I collagen deposition, both critical for a healthy healing process. The same authors also mentioned that a higher percentage of type I collagen probably relates to the high number of cells and blood vessels with high fiber cell activity and faster wound closure. Flavonoids, which appear to be the primary bioactive substances in *B. oleracea* extracts (10–20%), are thought to promote increased protein translation during the skin wound healing process (Miranda et al. 2024). Wang et al. (Wang et al. 2015) also revealed that intraperitoneal injection of collagen extracted from *Oncorhynchus keta* skin, at a dosage of 1.125 g/kg body weight, in an animal model following cesarean section in rats, exhibited enhanced wound contraction, increased collagen deposition, and angiogenesis. This facilitated improved wound healing post-cesarean section in rats (Wang et al. 2015). Nevertheless, a wide range of invertebrate marine organisms can serve as potential sources of collagen, which is extensively utilized as a biomaterial for skin regeneration, skincare, bone grafting, and dietary supplementation (Laasri et al. 2023).

Therefore, this study aimed to obtain the derivative CMC by chemical modification of CS, followed by cross-linking with MCP to obtain the complex CMC-MCP. In addition, the physicochemical properties of the above materials were determined and further evaluated by cellular modeling to assess their effects on cell migration in the HaCaT cell line. This initiative is expected to make a significant contribution to the development of an innovative biomaterial for wound healing applications.

## Materials and methods

### Materials

The dried CBs and cuttlefish mantles were provided by Hong Yu Foods Co., Ltd (Kaohsiung, Taiwan). CBs were stored in an expanded polystyrene container (half-filled with ice), then arrived at the laboratory within 30 min and were stored at −80 °C until used. The chemicals in this study were sourced and purchased from Sigma-Aldrich^®^ (Merck KGaA, Darmstadt, Germany) and were utilized without further treatment unless expressly stated otherwise. The HaCaT cell line was purchased from the American Type Culture Collection (ATCC; Manassas, Virginia, USA).

### Preparation of cuttlefish bones’ Chitosan (CS) and cuttlefish mantle collagen peptides (MCP)

CS and MCP were pre-treated and optimized for extraction individually according to the previously published methods by this team (Hazeena et al. 2022a, b). Specifically, CS in CBs was extracted using the conditions described in Hazecna et al. (2022a, b): a solid-liquid ratio of CBs to NaOH of 1:30 (*w/v*) in a water bath at 80 °C for 6 h, while the maximum average yield of 56.47% CS was obtained. In terms of MCP, the conditions described in Hazecna, Shih, et al. (Hazeena et al. 2022a, b) were used: cuttlefish mantles were mixed with a solid-liquid ratio of 1:20 (*w/v*) of HCl at pH 1.5, and 15 units (U)/mg of pepsin was added to obtain a maximum average extraction rate of 8.79% of MCP. The samples (CS and MCP) were individually packaged in double zipper bags and stored at 25 °C in a desiccator until used.

### Preparation of carboxymethyl Chitosan (CMC) and cross-linked cuttlefish mantle collagen peptides (MCP)

The CMC was prepared according to the method described by Bukzem et al. (Bukzem et al. 2016) with slight modifications. Briefly, 3 g of CS was mixed with 65 mL of isopropanol and stirred. Subsequently, 18.3 mL of 40% NaOH solution was added slowly over 15 min, followed by the addition of 6.9 g of monochloroacetic acid (an equal amount dissolved in isopropanol), and the reaction was carried out for 10.6 h (at 25 °C). Then, after filtration, the sediments were suspended in methanol and adjusted to a pH of 7.0 with acetic acid, filtered, and washed several times with 80% ethanol to remove excess material before drying (at 25 °C).

Next, the method of crosslinking CMC with MCP was based on the description of Cheng et al. (Cheng et al. 2020) with slight modifications. The 1% CMC and MCP (*w/v*) were prepared separately using 0.2 M, pH 6.0 phosphate buffer solution (PBS). Next, 0.35 g of 1-ethyl-(dimethylaminopropyl) carbodiimide (EDC) and 0.15 g of N-hydroxy succinimide (NHS) were added to 100 mL of CMC solution and stirred for 2 h. Then, the MCP solution was added at a ratio of 1:6 (*v/v*), which was stirred at 40 °C for 16 h. Afterward, the mixture was dialyzed in 5 L of distilled water for three days (the distilled water was changed every 8–12 h). Followed by 3 freeze-thaw cycles and then freeze-drying to obtain the cross-linked product CMC-MCP.

### Determination of degree of substitution (DS)

The DS determination of CMC-MCP was performed according to the method described by Cheng et al. (Cheng et al. 2020), while the principle was based on the calculated number of carboxyl groups (–COOH) that MCP replaces in the CMC repeating units. Briefly, standard curves were prepared using MCP (1.25, 2.5, 5, 10, and 20 mg/mL) by measuring the absorbance values at 200 nm, followed by the final calculation of DS (%) by the formula below.

1 $$\begin{aligned} & Degree\:of\:substitution\:(DS; {\%}) \\ & \quad =\frac{9800\times\:C}{920-46600\times\:C}\times\:100 \end{aligned}$$

Where, C is the MCP concentration in CMC-MCP (mg/mL).

### Crystalline structure analysis

The samples’ crystalline structure was determined following the method described by Huang et al. (2024) with slight modifications. The sample (100 mg) was analyzed using an X-ray diffraction (XRD) pattern (D5000, Siemens, München, Germany). The analysis was performed at an operating current of 40 mA and 15 kV with a Cu Kα X-ray source. The scanning speed was set to 0.06°/sec, while the scanning angle (2θ) ranged from 10° to 50°.

### Short-range ordered structure analysis

The sample was analyzed for short-range ordered structure using the method described by Lin et al. (2023a, b), with minor modifications. The 2 mg sample was mixed with 100 mg of dried potassium bromide (KBr) in a 1:50 weight ratio, producing 3 mm in diameter tablets. Subsequently, Fourier transform infrared spectroscopy (FTIR; FT-700 Series, HORIBA Group, Kyoto, Japan) was utilized to conduct wavelength scanning for analysis. The operational parameters included a wavelength range that extended from 4000 to 400 cm^− 1^, a resolution of 4 cm^− 1^, and a scanning frequency of 64 scans per unit time.

### Nuclear magnetic resonance (NMR) analysis

Following this team’s previous report, the samples were structurally characterized using ^1^H NMR measurements (Hazeena et al. 2022a, b). The experimental procedure involved dissolving a sample in a HOD solvent with 1% acetic acid-d_4_ (10 mg/mL) at 70 °C, then transferring 1 mL into an NMR tube (diameter of 5 mm). The spectra were performed using the 400 MHz NMR system (Unity Plus, Varian Medical Systems, Inc., Palo Alto, CA, USA). The chemical shift (δ) was reported in ppm units, while the splitting constant (J) was expressed in Hz. The measurement reference point was set to the residual solvent signal of D_2_O at a δ_H_ value of 4.80 ppm.

### Microstructure analysis

The microstructure analysis of the sample was performed using the method described by Yu et al. (2024) with slight modifications. Each sample (100 mg) was coated with a thin layer of gold before being examined under a field emission scanning electron microscope (SEM; S3000N, Hitachi, Ltd., Tokyo, Japan) at an accelerating voltage of 5 kV and 500× magnification.

### Evaluation of wound healing (in vitro)

#### Cell line culture

In this study, the HaCaT cell lines were cultured in Dulbecco’s modified Eagle’s medium (DMEM) containing 3.7 g/L sodium bicarbonate (NaHCO_3_), 10% fetal bovine serum (Gibco, Grand Island, NY, USA), and 100 U/mL penicillin-streptomycin (Gibco) in a humidified incubator (Panasonic Holdings Co., Osaka, Japan) at 37 °C, 5% CO_2_.

#### Cell viability (MTT method)

The cell viability (MTT method) was determined according to the method described by Huang et al. (2012) with slight modifications. The HaCaT cell line (1 × 10^5^/mL) was inoculated in 96 wells, added with different concentrations of CS (0–1600 µg/mL), MCP (0–6 mg/mL), CMC (0–400 µg/mL), and CMC-MCP (0–1600 µg/mL), and then cultured for 24 h. Afterward, the culture was incubated with DMEM medium containing 0.1 mg/mL 3-(4,5-dimethylthiazol-2-yl)-2,5-diphenyltetrazolium bromide (MTT) for 3 h. Next, remove the culture medium and add 100 µL of DMSO to dissolve the MTT-formazan product and shake for 50 min. Finally, the absorbance at 570 nm was measured using an ELISA reader (BioTek Synergy HTX, Agilent Technologies, Inc., Santa Clara, CA, USA). The following formula calculates the cell viability.

2 $$\begin{aligned} & Cellviability\left(\mathrm{\%}\right)\\ & \quad =\frac{Absorbance\:570\:nm\:of\:the\:Sample}{Absorbance\:570\:nm\:of\:the\:Contol}\times\:100 \end{aligned}$$

#### Cell migration rate

The cell migration methodology in this study was based on the approach described by Kishore et al. (Kishore et al. 2023) with minor modifications. Install the 2-well culture-insert (ibidi GmbH, Gräfelfing, Germany) into the 24-well, then inoculate the cell line (2 × 10^5^/mL) into the culture-insert wells. Allow the cells to attach for 24 h, then remove the culture insert and wash twice with PBS and different concentrations of samples CS (25, 50, and 100 µg/mL), MCP (0.2, 0.5, 1, 3, and 5 mg/mL), CMC (25, 50, 100, and 200 µg/mL), and CMC-MCP (25, 50, 100, and 200 µg/mL) were added to incubate for 24 h. Next, cell migration was observed and photographed using an automated microscope (LionHeart FX, Agilent Technologies, Inc.), and the photographs with the setting that the photographs would be taken every 6 h and removed after 24 h. The area was quantified using Image J software (bundled with Zulu OpenJDK version 1.52p, freely available, Image. sc Forum), and then the cell migration rate (also called the healed area of the wound) was calculated using the following formula:

3 $$\begin{aligned}Cellviability\left(\%\right) & =\left(\right(Areas\:of\:cellmigration\:at\:different\:time\:points \\ &\quad -The\:initial\:\left(0h\right)\:area\:of\:each\:group\left)\right) \\ & \quad /\left(The\:initial\right(0h\left)\:area\:of\:each\:group\right)\times\:100\end{aligned}$$

#### Gene expression

The cell lines were incubated on an ice bath to remove the culture medium and then washed twice with PBS. Next, the RNA extraction kit (GeneDireX Inc., Taoyuan, Taiwan) was used, following the manufacturer’s provided procedure. Briefly, 500 µL of GR Buffer 1 and 8 µL of β-peril ethanol were added and reacted for 10 min at 60 °C. Subsequently, 58 µL of GR Buffer 2 and 500 µL of chloroform were added and shaken vigorously, and centrifuged for 10 min (14,000 ×*g*, 4 °C). Next, transfer the supernatant to a new tube (1.5 mL DNA LoBind^®^ Tubes, Eppendorf Group, Hamburg, Germany), add an equal amount (1:1, *v/v*) of isopropanol, and mix uniformly. The reaction was transferred for 5 min on an ice bath to facilitate RNA precipitation and centrifuged again for 15 min (14,000 ×*g*, 4 °C). Afterward, the supernatant was removed, 1 mL of 70% ethanol was added to wash the residues, centrifuged again for 5 min (14,000 ×*g*, 4 °C) to remove the residual ethanol, allowed the RNA to air-dry at 25 °C, and finally, DEPC-H_2_O was added to re-dissolve the precipitates. Next, the absorbance values of the samples at wavelengths of 260 and 280 nm were determined, while the total RNA qualities of the samples were calculated (the ratio of ABS 260/280 nm should be within the range of 1.9–2.1).

The next step was performed using a cDNA kit (GenDireX, Inc.) and following the manufacturer’s operating procedure. Add 10 µL of RNA, 1 µL of Oligo (dT) 20, and 1 µL of dNTP mix to a 0.2 mL tube. Add sterile water to a total volume of 13 µL and mix evenly. Then, the tubes were heated in a dry bath at 65 °C for 5 min, shaken, and then placed in an ice bath for 5 min. Afterward, add 4 µL of 5 × 1st strand buffer, 1 µL of DTT, 0.25 µL of RiboINTM RNase inhibitor, and 1 µL of GScript RTase. Then, add sterile water to achieve a total volume of 20 µL and shake to mix well. React in a dry bath (50 °C) for 1 h. Finally, heat at 70 °C for 15 min before obtaining the single-stranded cDNA solution, which should be stored at −20 °C until use.

In the 96-well dish for polymerase chain reaction (PCR), 5 µL of 2× SYBR Fast MM, 0.5 µL of 20× Target gene primer, 2.5 µL of ddH_2_O, and 2 µL of 10 ng cDNA were added. Afterward, reaction and fluorescence quantification were performed using a Real-time PCR system (LightCycler^®^ 480, Roche Life Science, Basel, Switzerland) (detailed conditions as Table A1). In addition, human Glyceraldehyde-3-Phosphate Dehydrogenase (GAPDH) was used as a quality control group for relative quantification in this study. The gene-specific primers for each gene are shown in Table A2.

### Statistical analysis

Three replications of each trial were performed in this study, and all data were expressed as mean ± standard deviation. All results were analyzed using one-way analysis of variance (ANOVA) with Statistical Package for the Social Sciences (SPSS) Software (version 12, International Business Machines (IBM) Co., Armonk, New York, USA). Post-hoc comparisons were analyzed using Duncan’s Multiple Range or Tukey’s method, which indicated significant differences, where *p* < 0.05, and differences between groups were compared.

## Results and discussion

### Variations in physicochemical properties by modification and cross-linking induce cuttlefish bone Chitosan (CS) and the complexes

#### Degree of substitution (DS)

**Fig. 1:**
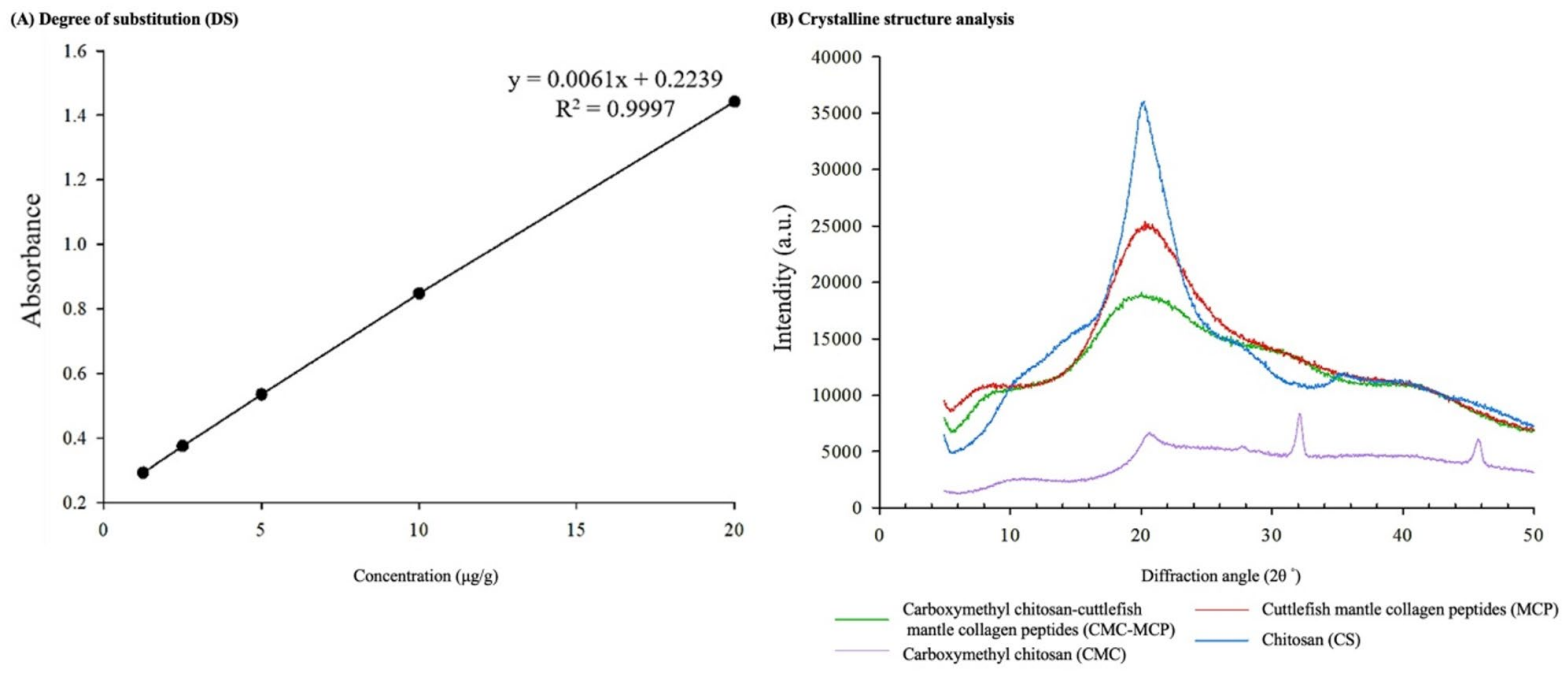
Effects of modified cross-linking of chitosan (CS) derived from Cuttlefish bone (CB) on **A** degree of substitution (DS) and **B** crystalline structure

This study used CMC (modified CS), EDC, and NHS covalently conjugated with the MCP to form CMC-MCP derivatives, while the DS (%) revealed through the standard curve analysis (Fig. 1A) was calculated to be 61.4 ± 9.2%. The formation of cross-linking substances through substitution differences has been hypothesized to arise from the potential stereo hindrance effect (Cheng et al. 2020), coupling reaction (Zhong et al. 2019), and electrostatic repulsion (Hua et al. 2016). It has been documented that employing diverse ratios of reaction matrices in the cross-linking process can lead to varying DS (Cheng et al. 2020). The beneficial effect of high polymer DS on wound healing potential has been reported, and the deposition of collagen is facilitated by arginine-modified CS-polyvinyl alcohol hydrogel (Hasnain et al. 2023).

In addition, it has been reported that the amine (C2-) and hydroxyl (C3- and C6-) functional groups in CS can be subjected to the introduction of carboxymethyl, resulting in CMC effectively enhancing its solubility (Elnaggar et al. 2024; Geng et al. 2023; Samadi et al. 2016). It is worth noting that precise pH control serves a crucial role in EDC/NHS conjugation (D’Este et al. 2014; Guo et al. 2024). Specifically, a pH of 3.5–4.5 balances EDC’s effective activation with the amines’ protonation (non-nucleophilicity at high pH) (Guo et al. 2024). It has been reported that manipulation of pH can effectively regulate the solubility of related compounds across diverse pH environments (Guo et al. 2024), offering a wide range of applications. Interestingly, Horo et al. (2019) reported that EDC and NHS served as catalysts to initiate the covalent conjugation and cross-linking of low molecular weight CS with 5-fluorouracil to form more water-soluble hydrogels via the –COOH group, with controlled release achieved by slicing under 365 nm UV-A radiation. Moreover, substituted chemical modifications have been reported that potentially enable unique pharmacological properties (bactericidal, antibacterial, and antiviral), stimulation of the complementary system (platelet activation and inducing erythrocyte aggregation), bioavailability, and biodegradability with no toxicological hazards (Elnaggar et al. 2024). Therefore, DS can be modified by varying the ratio of substrates, temperatures, and times in the reaction to achieve the required modification (cross-linking) effects.

#### Crystalline structure

This study revealed a pronounced peak at 21° in all samples, with the weakest intensity observed in the CMC sample (Fig. 1B). This observation can be attributed to intramolecular and intermolecular hydrogen bonding structures within the crystalline region of CMC, which also contribute to its limited solubility (Wang et al. 2020a, b). Despite being reported as a derivative of CS that inherits its advantages, CMC is prone to chemical modification and further processing due to the abundance of reactive groups (hydroxyl and amino groups) in its molecular chain (Yang et al. 2024a, b). In addition, the peaks of all the functional groups in the CS-modified CMC were comparatively less pronounced and exhibited weaker intensities than the other samples. These phenomena implied low degrees of crystallinity, which can be attributed to the O-substitution of hydrogen bonds in hydroxyl and amino groups with carboxyl groups. Consequently, the reduced number of hydrogen bonds contributes to diminished crystallinity (Cheng et al. 2020). It has also been reported that a negative correlation exists between the degree of crystallinity of CM-CS and the degree of cross-linking (DS) (Katugampola et al. 2014). The peak intensity of MCP at 21° was also less than that of CS, while the CS samples reportedly exhibited a broadening of the peak band at 2θ = 20–21° in cases where other substances were grafted or cross-linked (Aguirre-Pranzoni et al. 2023). This was attributed to hydrolysis by pepsin during preparation, which selectively cleaves the peptide sequences at the end of collagen fibers, weakening the intermolecular interactions between collagen molecules, and thus increasing the distances between the molecular chains (Chen et al. 2019a, b). This results in the alteration of the initial crystalline structure (Chen et al. 2024a, b). However, the peak intensity of CMC-MCP at 21° was also lower than that of MCP and CS, as observed following the cross-linking of CMC with MCP, but the peak range was widened. The observed phenomenon was ascribed to the molecular interactions between CMC-MCP, which induced alterations in the crystalline structure of CMC-MCP (Cheng et al. 2020). Specifically, these alterations involved the original hydrate structure of CS through the incorporation of massive side groups or cross-linking, which disrupted the molecular interactions between the chains and was accompanied by a loss of crystallinity in the CS (Aguirre-Pranzoni et al. 2023; Ait Hamdan et al. 2024).

#### Short-range ordered structures

**Fig. 2:**
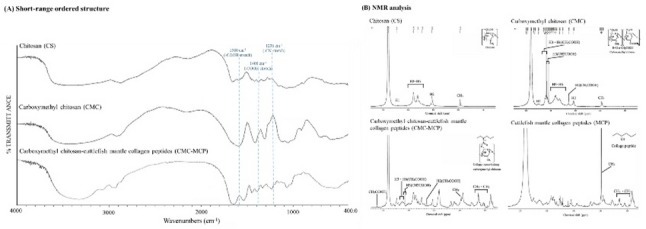
Effects of modified cross-linking of chitosan (CS) derived from Cuttlefish bone (CB) on **A** short-range ordered structure and **B** ^1^H nuclear magnetic resonance (NMR) analysis

The results of this study indicated that the short-range ordered structures of the three samples are similar, with differences primarily reflected in the characteristic absorption peaks (Fig. 2A). Compared to CS, both CMC and CMC-MCP exhibited broader and more intense peak bands in the 3000–3300 and 1560–1645 cm^− 1^ ranges, corresponding to the stretching vibrations of N–H in amides and O–H, while these findings align with those reported by Hazeena et al. (2022a, b), Hasnain et al. (2023), and Lin et al. (2023a, b). The peak of amide I at 1650 cm^− 1^ corresponds to C=O stretching, while the peak of the amino group at 1500 cm^− 1^ corresponds to –NH bending) (Hazeena et al. 2022a, b; Ait Hamdan et al. 2024; Lin et al. 2023a, b; Deng et al. 2021; Mondéjar-López et al. 2024). The 1400 and 1580 cm^− 1^ peaks represent antisymmetric vibrations of the CMC-COOH (Dobaj Štiglic et al. 2021; García et al. 2024; Yang et al. 2024a, b; Zhang et al. 2024a, b), while 1322 cm^− 1^ represents the C-N stretching vibration of the residual n-acetyl group (Hu et al. 2019). Notably, the difference between CMC and CMC-MCP compared to CS showed peaks at 1407 cm^− 1^, which were related to the symmetric stretching vibration of –COO (Hu et al. 2019; Bukzem et al. 2016; García et al. 2024). However, the peaks of CMC-MCP in the above wavelength bands were weakened, indicating the reduction of carboxyl groups following the successful cross-linking of MCP with CMC. At 1236 cm^−1^, the C−H of the amide region III in CMC exhibited a distinct absorption peak compared to the other samples (Hu et al. 2019; Lin et al. 2023a, b; Khan et al. 2023). In addition, the differences between the CMC and CMC-MCP absorption peaks at 1029 cm^− 1^ also suggested that the cross-linking substitution occurred primarily at the C6 position (Hu et al. 2019). Notably, the EDC/NHS system effectively connected the amine group of CMC with the carboxyl group of MCP, which facilitated the formation of short-range amide bonds, and the results were agreed with those reported by Goodarzi et al. (Goodarzi et al. 2019). However, the other evidence of the variations with the ^1^H NMR in the next section can also be consistently supported.

#### ^**1**^**H NMR spectra**

The ^1^H NMR results of this study revealed that the characteristic peak positions of CS, CMC, and CMC-MCP were identical (Fig. 2B). Specifically, the distinctive peak at 2.0 ppm represented the CH_3_ group, while the peak near 3.18 ppm indicated the –CH group attached to the C2 of CS (Hu et al. 2019; Ait Hamdan et al. 2024). In addition, signal values ranging from 3.4 to 4.0 ppm corresponded to the CH_2_COOH to C3–6 of CS (Hu et al. 2019; Hazeena et al. 2022a, b; Bukzem et al. 2016; Aguirre-Pranzoni et al. 2023; Tang et al. 2016), whereas signals at 4.57 and 4.86 ppm represent hydrogen on C1 (Hazeena et al. 2022a, b). Notably, the CMC signals between 4.20 and 4.34 ppm correspond to the two hydrogen signals of the C6 carboxymethyl groups bonded to the hydrogen signals of the C3 carboxymethyl groups. Following derivatization, a significant increase in carboxymethyl group content was observed, as evidenced by the intense signal values at 4.21 ppm. In addition, the 4.12–4.19 ppm ranges were ascribed to the presence of hydrogen-bonding to the carboxymethyl group on C3, whereas the 3.25–3.45 ppm ranges corresponded to the signal arising from bonding between the carboxymethyl group and C2. In contrast, the signal at 4.09 ppm represents the O-CMC signature signal (Chen et al. 2004), namely, the –CH_2_COOH group substituting for the –OH functional group in the CS glucose unit, effectively addressing the inherent solubility issue of CS, which was also reflected in its appearance and microstructure (Chen et al. 2024a, b). Notably, the cross-linking of CMC with MCP results in distinctive peaks at 0.9–1.4 ppm, presumably associated with the involvement of CH_2_ and CH_3_ groups. In parallel, the characteristic peak at 4.21 ppm for CMC-MCP exhibits a significant reduction compared to that of CMC, which can be attributed to the decreased number of carboxyl groups resulting from the bonding of MCP with CMC. Moreover, the CMC-MCP exhibited a distinctive peak at 5.21 ppm, attributed to the hindered rotation of N–CO and resulting in spin conformer isomers (Hu et al. 2019), while an identical peak signal was also observed in the MCP spectrum.

#### Appearance and microstructure

**Fig. 3:**
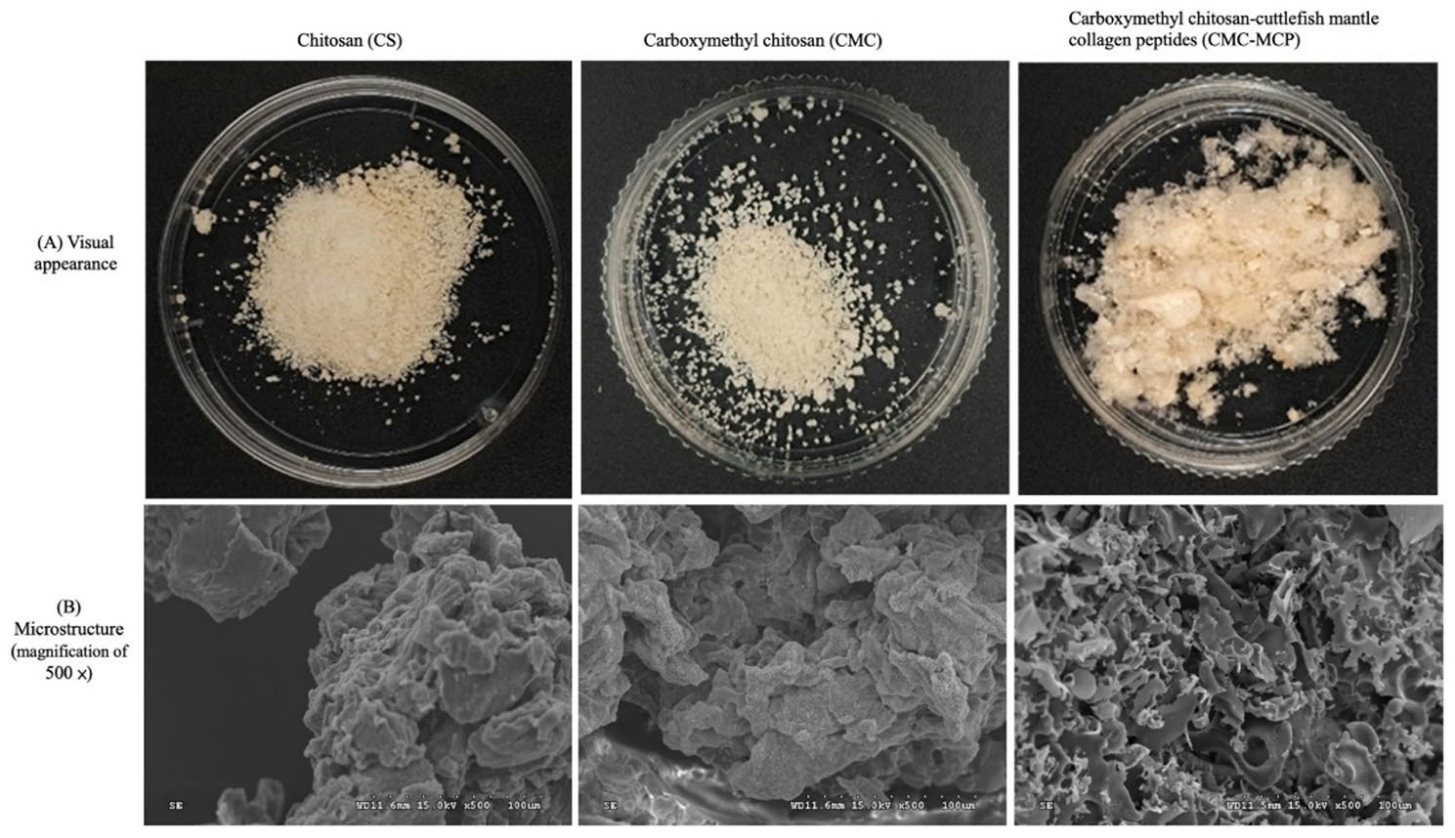
The appearance and microstructure of modified cross-linking of chitosan (CS) derived from Cuttlefish bone (CB)

The visual appearance (Fig. 3A) of the three samples in this study revealed that the alkaline-extracted CS presented light yellow particles of varying sizes, while the CMC exhibited a similar distribution of particles, albeit with greater elasticity and a lighter color. In contrast, the CMC-MCP cross-linkage resulted in loose and fragile, light yellow cotton wool that was soft, hygroscopic, and exhibited excellent water solubility. This elevated water solubility can be attributed to the fact that MCP primarily consists of many peptides with small molecular weights, while the exposure of multipolar residues to water contributes to more hydrogen bond formation (Hu et al. 2017). Moreover, the microstructure of the sampled CMC (magnification of 500×) exhibited a more blocky stacked morphology and a rougher appearance compared to the CS (Fig. 3B). It also implies that the structure contributes to surface area enhancement, namely, increased solubility due to easier interaction with water molecules. The formation of –COOH groups was postulated to account for this phenomenon (Chiu et al. 2007; Jeon et al. 2001). Conversely, CMC-MCP exhibited a distinctive three-dimensional loose porous morphology with irregularly distributed pores that were more dispersed rather than uniformly arranged (Liu et al. 2024; Zhang et al. 2024a, b; Zhao et al. 2024). This occurrence was ascribed to the interplay between hydrogen and amide linkages within CMC and MCP (Cheng et al. 2020). Notably, this property’s structure facilitates hemostasis and promotes wound healing by effectively absorbing substantial blood and leachate at the injury site (Zhang et al. 2024a, b).

### Effects of carboxymethyl Chitosan (CMC) cross-linked cuttlefish mantle collagen peptides (MCP) on wound healing (in vitro)

**Fig. 4:**
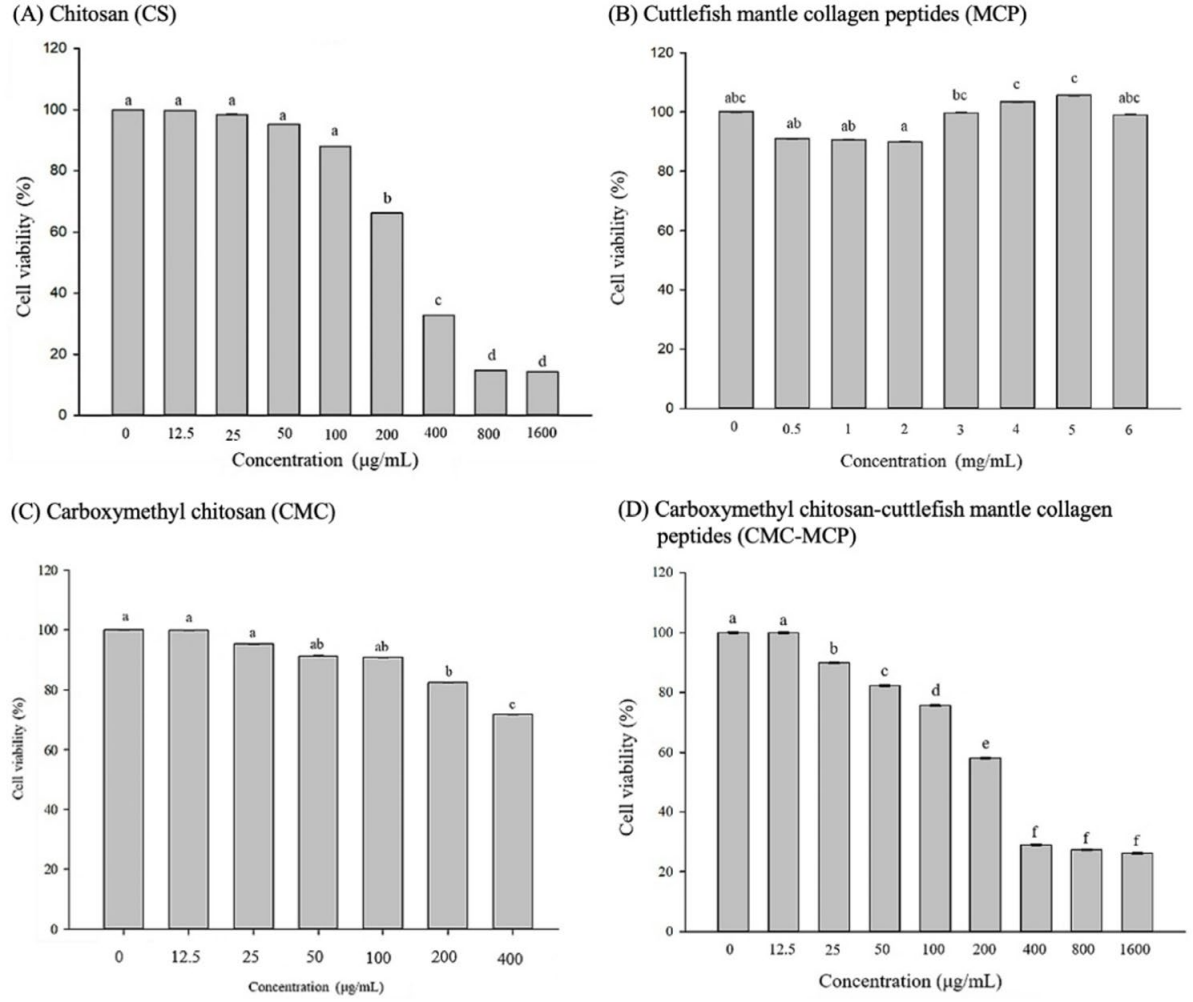
Effects of **A** chitosan (CS) derived from Cuttlefish bone (CB), **B** cuttlefish mantle collagen peptides (MCP), **C** carboxymethyl chitosan (CMC), and **D** CMC-MCP on cell viability

Commonly, it is crucial for new materials intended for use in organisms to possess low cytotoxicity, with cell viability exceeding 80%, as this is favorable for subsequent advancements and applications (Chelminiak-Dudkiewicz et al. 2024; Gruber and Nickel 2023; Thangaraju and Varthya 2022). The optimal concentration for each sample group, ensuring no apoptotic effects, was confirmed for the HaCaT cell line viability using MTT prior to the functional evaluation of wound healing in this study. The results of this study showed that concentrations of CS exceeding 100 µg/mL caused varying degrees of growth inhibition in HaCaT cell lines (*p* < 0.05) (Fig. 4A). Thus, a concentration below 100 µg/mL was preferred for the subsequent functional evaluation trial. The 0.5–6 mg/mL MCP concentration showed no adverse effect on the HaCaT cell lines’ viability (Fig. 4B), while some concentrations even promoted their growth. However, it has been reported that the enriched amino acid residues in MCP may provide suitable conditions for the migration of induced HaCaT cell lines (García et al. 2024; Hu et al. 2017). In contrast, the trends of CMC and CMC-MCP were similar to those of CS (*p* < 0.05) (Fig. 4C and D). It is worth mentioning that CMC containing hydrophilic carboxymethyl groups has been reported as non-toxic and can be fabricated into dressings that remain moist in the wound environment (Lin et al. 2020; Taokaew et al. 2023). Furthermore, it has been reported that dialysis effectively eliminates EDC and NHS (covalently conjugated catalysts), with a concentration below 0.3 mol/L considered biologically safe (Hua et al. 2016), which was consistent with the results of this study. Therefore, functional evaluation experiments were conducted with concentrations below 100 µg/mL.

#### Scratching experiment to evaluate cell line migration proficiency

**Fig. 5:**
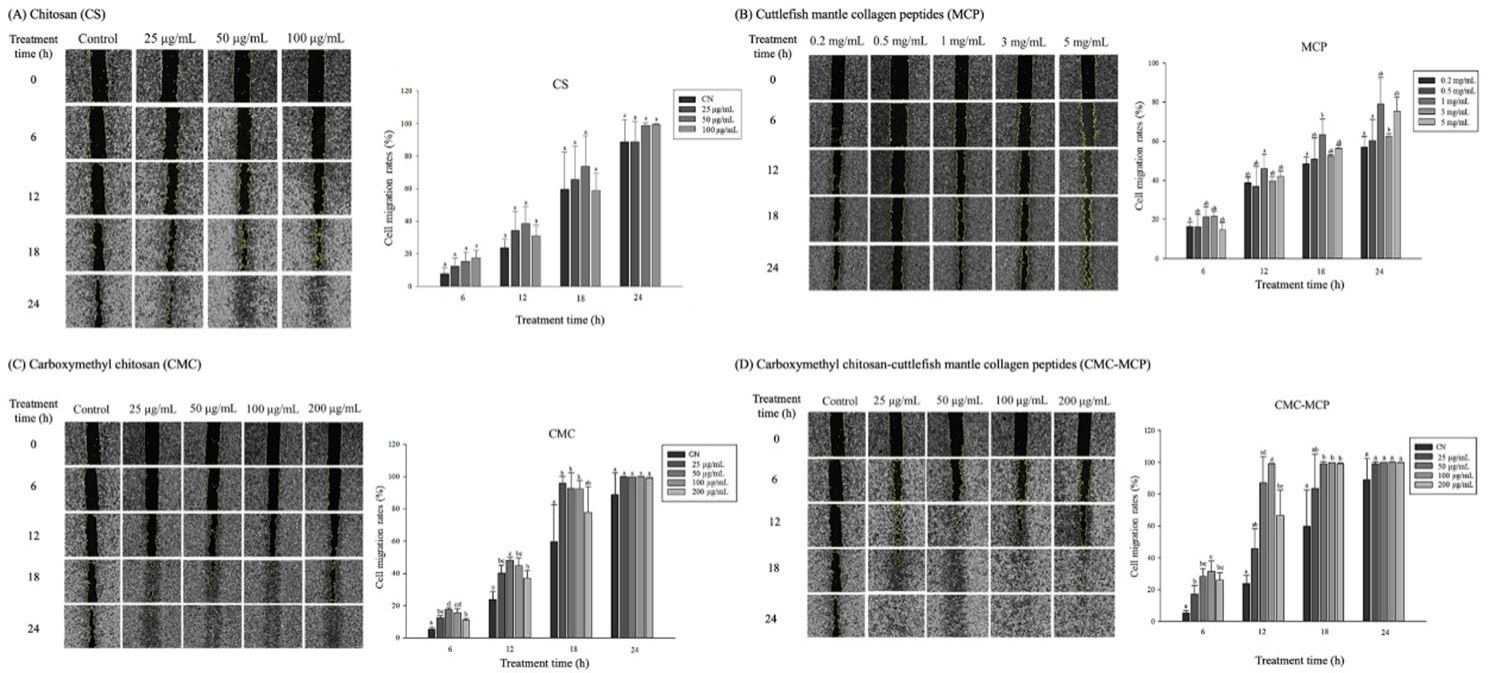
Effects of **A** chitosan (CS) derived from Cuttlefish bone (CB), **B** cuttlefish mantle collagen peptides (MCP), **C** carboxymethyl chitosan (CMC), and **D** CMC-MCP on the scratching experiment to evaluate cell line migration proficiency

This study showed no significant difference in CS compared to the control group despite a certain degree of dose dependence on cell migration rate (Fig. 5A). It is worth mentioning that studies have reported that CS surface charge with many cationic sites may adversely affect cell growth and attachment (Linju and Rekha 2023); namely, electrostatic ionic interactions between negatively charged cell membranes and cellular components and positively charged CS amino groups (Linju and Rekha 2023). During the process of skin damage, the pH value shifts from the slightly acidic state (pH 4.0–6.0), characteristic of healthy skin, towards a more neutral pH of 7.4 (Mutlu et al. 2022). However, the optimal pH range for the proliferation of keratinocytes and fibroblasts is between pH 7.2 and 8.3 (Mutlu et al. 2022). The same authors also indicated that while a slightly acidic pH was beneficial for wound healing by controlling infection and enzyme activity (such as zinc-dependent enzymes).

Regarding MCP, this study’s results showed that MCPs at 1 and 5 mg/mL were significantly (*p* < 0.05) associated with the promotion of cell migration in HaCaT cell lines compared to the control group at 18 h, respectively (Fig. 5B). The findings align with those reported by Lin et al. (Lin et al. 2021) and Jridi et al. (Jridi et al. 2015), indicating that collagen gel derived from *Sipunculus nudus* or cuttlefish skin can expedite wound contraction and hold promise as a potential wound-healing agent. However, the CS-modified CMC significantly enhanced the cell migration rate of HaCaT cell lines from 12 to 24 h (Fig. 5C), yet there was no significant difference at 24 h compared with the control. The study conducted by D. Wang et al. (Wang et al. 2020a, b) revealed that CMC exhibits the potential to enhance the proliferation of human skin fibroblasts through upregulation of α-smooth muscle actin (α-SMA) and transforming growth factor (TGF)-β1 expression in a cellular model. Furthermore, it demonstrated remarkable efficacy in promoting epithelialization and facilitating optimal wound healing outcomes in an animal model (Wang et al. 2020a, b). Moreover, CMC-MCP treatments exhibited a significant (*p* < 0.05) increase in the migration rate of HaCaT cell lines from 6 h as time passed, which was higher than the control group (Fig. 5D). The migration rate achieved 100% within 12 h when exposed to a CMC-MCP concentration of 100 µg/mL, indicating its potential efficacy in wound healing. Notably, the incorporation of gelatin in either CS or CMC cross-linking has been documented to facilitate wound healing while maintaining biodegradability and biocompatibility (Huang et al. 2013; Xu et al. 2018). Moreover, Naderi Gharahgheshlagh et al. (Naderi Gharahgheshlagh et al. 2024) reported that wound-healing scaffolds applying exopolysaccharide (*Rhodotorula mucilaginosa* sp.) conjugated to collagen (rainbow trout skin) promoted fibroblast proliferation and dissemination, primarily attributed to the exopolysaccharide’s antioxidant properties. In addition, Gao et al. (Gao et al. 2020) reported that leucine-rich α-2-glycoprotein-1 (LRG1) expression remained significantly elevated during the early stages of wound healing, promoting enhanced migration of HaCaT cell lines and forming a new epidermal barrier at the wound edge. This ultimately accelerates wound repair (Gao et al. 2020). Notably, it has been demonstrated that in the presence of TGF-β1, LRG1 has a mitogenic effect on endothelial cells, promoting angiogenesis (Wang et al. 2013). In addition, the excessive presence of cellular free radicals in injured tissues can impede the wound-healing process by inducing oxidative damage to cells (Yang et al. 2024a, b). Nonetheless, the CS utilized in this study has demonstrated commendable antioxidant capabilities (Hazeena et al. 2022a, b), implying an additional potential advantage offered by CMC-MCP alongside its ability to facilitate cell migration through collagen. In addition, incorporating naturally bioactive substances exhibiting antioxidant activity into the dressing has also demonstrated favorable outcomes in promoting expedited cell proliferation and wound healing (Chelminiak-Dudkiewicz et al. 2024; Saha et al. 2023). Another possible explanation might be that CMC-MCP’s functional groups, wettability, and moderate surface roughness stimulate the adhesion and proliferation of HaCaT cell lines (Khan et al. 2023). Specifically, binding sites in the form of the arginine-glycine-aspartate sequence of MCP with amino groups in the CMC scaffold promoted the behavior of the cells described above (Khan et al. 2023). Therefore, based on the above results, CMC-MCP obtained through CS modification and cross-linking exhibited a satisfactory ability to facilitate the migration of HaCaT cell lines, suggesting its potential application for promoting wound healing (Ciftci and Özarslan 2024; Yang et al. 2024a, b). Moreover, it has been reported that incorporating naturally derived botanical extracts into CMC or utilizing nanocarriers to regulate the release of bioactive proteins (such as TGF and vascular endothelial growth factor) and peptides could serve as a viable approach for promoting wound healing (Baran 2024).

#### Gene expression of wound healing

**Fig. 6:**
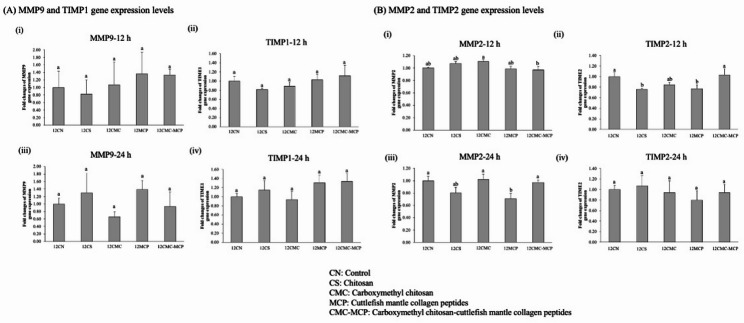
Effects of treating HaCaT cells with various samples [chitosan (CS) derived from Cuttlefish bone (CB), carboxymethyl chitosan (CMC), cuttlefish mantle collagen peptides (MCP), CMC-MCP) for 12 and 24 h on the gene expression of **A** matrix metalloproteinase (MMP) 9 and tissue inhibitors of metalloproteinase (TIMP)-1, and **B** MMP2 and TIMP-2

This study on the gene expression of HaCaT cell lines related to migration and wound healing in different treated samples showed that CMC, MCP, and CMC-MCP treatment for 12 h showed a trend of increased MMP-9 gene expression (Fig. 6A i). Yet, they were not significantly different compared to the control group. Specifically, the highest level of MMP9 gene expression was observed following CMC-MCP treatment; namely, it possessed a stronger ability to promote cell migration (consistent with the description in the previous section). This phenomenon was attributed to the fact that MMPs (2 and 9) can degrade the extracellular matrix (An et al. 2021; Ayuk et al. 2016; Tsilingiris et al. 2025), while the degradation facilitates keratinocyte mobilization, which is crucial in the early stages of wound healing (Caley and Martins 2015). It is worth mentioning that hydrophilic samples contribute to the diminished hydrophobicity of keratin, which may facilitate base membrane disassembly and cell migration (Baburao et al. 2024). Moreover, Tsilingiris et al. (Tsilingiris et al. 2025) documented disruptions in the neutrophil extracellular traps-fibroblast crosstalk in primary human skin fibroblasts (obtained from diabetic patients) during wound healing, wherein neutrophils enhanced the expression of interleukin (IL)-8 in neutrophils and stimulated the production of MMP-9 in neutrophils. In addition, the same trend as above was observed in TIMP-1 gene expression (Fig. 6A ii), and there were insignificant differences between groups. This phenomenon was suggested to be attributed to the fact that the treatment of these samples, apart from activating MMP-9 expression, may also be involved in the negative regulation of its activity. Specifically, this mechanism would facilitate maintaining tissue stability and avoid excessive matrix degradation (Mavridis et al. 2010). After 24 h of treatments, the expression trends of MMP-9 and TIMP-1 (Fig. 6A iii and iv) across all groups were consistent with the above results. Similarly, the expression levels of MMP-2 and TIMP-2 in HaCaT cell lines treated with different samples for 12–24 h (Fig. 6B i–iv) showed similar trends to those above, with no statistically significant differences compared to the control group. This phenomenon can be ascribed to the temporal alterations in cell lines and the wound healing expression patterns of genes (MMP2 and TIMP-2) related to tissue remodeling (Guo et al. 2020). It has been reported that TIMPs play a crucial role in promoting wound healing, which is achieved by inhibiting the expression of MMPs and inflammatory cytokines in epithelial cells (Folorunso et al. 2025). Kishore et al. (2023) reported that the use of nanocomposites with keratins would be used to induce natural keratinocytes through the expression of MMP2 to improve wound healing. Meanwhile, the same authors also note that the silver nanoparticles in their materials are attracted to active sites that interfere to some extent with metalloproteinases. Summer et al. (2025) also reported that treatment in diabetic mice with *Bergenia ciliata* extract-nanoparticles-loaded salvia hispanica hydrogel facilitated wound healing and restoration of gene expression levels of MMP2 and TIMPs.

Therefore, these results indicated that the treatment groups initially promoted cell migration and then progressed to the remodeling and stabilization of wound healing. In particular, the treatment of CMC-MCP was demonstrated to be highly effective in enhancing the wound healing repair modulation potential of HaCaT cell lines. Based on the above results, the present study demonstrates that CMC-MCP contributes to the up-regulation of MMP2 and 9 expression during wound healing (Kishore et al. 2023). This also implies that optimal wound care is critical to establishing a favorable cellular tissue regeneration and repair environment (Baniasadi 2025). Moreover, it can potentially be cost-effective and conveniently available to replace existing commercial products (porcine, bovine, human cadaver skin, or other derivatives) (Saha et al. 2023). However, a limitation of this study is the need for further animal experiments (either in rodents or higher-level animals) to gain a more in-depth understanding of the CMC-MCP and to inform future clinical trials.

## Conclusions

This study revealed that in the cellular mode, 50 µg/mL of the CMC-MCP complex can promote cellular migration, indicating a role in wound healing. However, the limitation of this study refers to the need for further confirmation of the expression of genes for inflammation and angiogenesis, etc., before assessing their functionality (tissue regeneration, inflammation, angiogenesis, and scar elimination, etc.), biodegradation, and biocompatibility through animal and clinical trials. It is also necessary to explore the synergistic use of other physical methods (such as nanomaterials, cold plasma, γ-ray irradiation, etc.) for cross-linking in the future, anticipating the reduction of the dependence on organic solvents. Altogether, this study demonstrated and emphasized that the CMC-MCP complex, obtained by cross-linking CMC and MCP, is a promising dressing that enhances wound healing.

## Data Availability

Data supporting this study’s findings are available upon reasonable request from the corresponding authors.

## Appendix

See tables 1 and 2.

**Table 1:** Conditions of operation for Real-time polymerase chain reaction (RT-PCR)

Program	Temperature (°C)	Time (sec)	Cycle
1	95	120	× 1
2	95	5	× 40
	60	30	
3	95	10	× 1
	65	60	
	97	1	

**Table 2:** Gene-specific primers for human genes used in Real-time PCR

Gene	Primer sequence (5'-3')
Glyceraldehyde-3-Phosphate Dehydrogenase (GAPDH)	F- CTCCTCCACCTTTGACGCTG R- CTCCTTGGAGGCCATGTGG
Matrix metalloproteinase (MMP)-2	F-CATGCGGAAGCCAAGATGT R-TCCAGGTCAGGTGTGTAACCAA
MMP-9	F-TCGCGTGGATAAGGAGTTCTC R-GGAAACTCACACGCCAGAAGA
Tissue inhibitor of metalloproteinase (TIMP)-1	F-CGTTGGAGGAAAGAAGGAATATCTC R-ACAGAGGGTGATGTGCATCTTG
TIMP-2	F-ATGCACAGTGTTTCCCTGTTTATC R-GGTCCGTCCACAAGCAATG
